# Medial pulvinar stimulation for focal drug-resistant epilepsy: interim 12-month results of the PULSE study

**DOI:** 10.3389/fneur.2024.1480819

**Published:** 2024-12-10

**Authors:** Francesca Pizzo, Romain Carron, Virginie Laguitton, Audrey Clement, Bernard Giusiano, Fabrice Bartolomei

**Affiliations:** ^1^Department of Epileptology and Cerebral Rhythmology, APHM, Timone Hospital, Marseille, France; ^2^Aix Marseille Univ, INSERM, INS, Inst Neurosci Syst, Marseille, France

**Keywords:** deep brain stimulation, neuromodulation, quality of life in epilepsy, seizure reduction, epilepsy comorbidities

## Abstract

**Objective:**

This study aims to evaluate the efficacy and safety of deep brain stimulation (DBS) of the medial pulvinar nucleus (PuM) in reducing seizure frequency and addressing comorbidities in patients with drug and vagal nerve-resistant focal epilepsy.

**Methods:**

This is an open-label prospective treatment trial with a planned enrollment of 12 patients suffering from medically refractory epilepsy (Clinical trial gov NCT04692701), for which the interim 12-month post-implantation results for the first 6 patients are being reported. Inclusion criteria were focal epilepsy not suitable for or after failed surgical intervention and previous failure of neurostimulation therapies (vagus nerve stimulation or anterior thalamic nucleus DBS). Evaluations included seizure diaries, neuropsychological assessments, and scales for depression, anxiety, quality of life, and seizure severity. PuM DBS was performed using ROSA robotic assistance, with follow-ups every 3 months for 1 year.

**Results:**

Out of six patients, five completed 1-year follow-up (one patient died prematurely). A non-significant trend toward seizure reduction was observed at 6 months, becoming more pronounced at 1 year (mean reduction: 45%; responders: 2/5). Seizure severity significantly improved (*p* = 0.02), with a reduction in the NHS3 scale scores. Quality of life improved significantly at 1 year (*p* = 0.03). Psychiatric assessments indicated a non-significant trend toward improvement in depression (mean improvement: 26%) and anxiety (mean improvement: 20%) scores. Neuropsychological testing showed stable or improved cognitive performance in three out of five patients. Adverse events included one case of cerebral hemorrhage, one infection leading to device removal, and one possible SUDEP.

**Significance:**

Preliminary results suggest that PuM DBS may offer a promising therapeutic option for reducing seizure severity and improving quality of life and cognitive functions in patients with drug-resistant epilepsy. Despite the small sample size and the presence of serious adverse events, the findings warrant further investigation with larger cohorts to confirm these trends and optimize the treatment protocol.

## Introduction

Epilepsy affects approximately 1% of the general population, and one-third of patients are drug-resistant (1, 2). In some cases (20%), surgical resection can be offered, effective in approximately 50–70% of cases (1, 2). There is, therefore, a large population of patients for whom resective surgery is contraindicated or insufficiently effective and to whom palliative therapies are offered.

Neuromodulation represents an increasingly attractive treatment option for those patients (3). Neuromodulation can take the form of nerve stimulation, such as vagus nerve stimulation (VNS) or intracranial stimulation, such as responsive neurostimulation (RNS) or deep brain stimulation (DBS) (4).

VNS is the most commonly used of palliative therapies but has a 50% failure rate, and only 3–5% of patients are seizure-free (5). The stimulation of the anterior nucleus of the thalamus (ANT) has shown a seizure reduction of approximately 40% (6). Different brain targets for DBS have been tested, including the centromedian thalamus, hippocampus, motor cortex, caudate nucleus, and subthalamic nucleus, with a general overall efficacy in seizure reduction of approximately 35–60% (3). Except for anterior thalamic nucleus stimulation (6, 7), no randomized control trials exist in the other targeted deep nuclei stimulation.

It is crucial to note that patients with drug-resistant epilepsy frequently suffer (up to 60% of cases) from cognitive and psychiatric comorbidities (8). Depression is a significant factor affecting the quality of life of these patients. The VNS has a positive effect on depression (9), while the DBS of the anterior nucleus of the thalamus could have a more negative effect (10).

Pioneering studies using stereoelectroencephalography (SEEG) (11, 12) had already shown the participation of the thalamic median pulvinar nucleus (PuM) in the propagation and amplification of temporal seizures (11) and its possible role in their arrest (13). PuM is also involved in interictal activities, being more pronounced in patients with a worse surgical outcome (14). The PuM has extensive cortical connections (15), particularly with temporoparietal associative areas, and it is involved in seizures arising from different brain areas (16). PuM involvement in extratemporal lobe seizures was also previously shown (11, 12, 16). This nucleus, importantly, plays a role in the ictal loss of awareness (LOA) (17). The degree of LOA during temporal seizures was found to correlate with the amount of synchronization in thalamocortical systems. Interestingly, thalamic stimulation of the ANT (18) and the PuM (19) has been shown to reduce SEEG signal synchrony in responders to stimulation. Similar connectivity modifications were also found in responders to VNS (20). Recent data showed that stimulation of the PuM is well tolerated and can effectively reduce the duration and severity of attacks (21, 22).

From a surgical perspective, PuM is more readily accessible than the anterior nucleus of the thalamus in the setting of SEEG (23). In the case of DBS, the ANT’s proximity to the lateral ventricle and its vascular anatomy, including the nearby choroid plexus and thalamic-striate veins, can make it difficult to target precisely, with some evidence suggesting the trans-ventricular approach. In comparison, the PuM may present a less challenging target to access.

We, therefore, proposed a pilot study (Clinical trial gov NCT04692701) to evaluate the impact of PuM deep brain stimulation on the frequency of epilepsy seizures and comorbidities (psychiatric, cognitive) in a population of patients with focal epilepsy considered inoperable (or failing prior surgery) and in patients who failed neurostimulation therapies as VNS or thalamic anterior nucleus DBS.

## Materials and methods

### Study design

This is an open-label prospective pilot treatment trial involving patients with medically refractory epilepsy. In this trial, patients serve as their own internal controls and are followed for 24 months after implantation. The interim results after 1 year for the first six patients are being reported.

The final sample size of the trial will be 12 patients. It has been estimated that the average seizure frequency before inclusion in the study ranges between 8 and 20 seizures/month, though this estimate is subject to inaccuracy because of the small sample size. The standard deviation is estimated to be between 4 and 13. The goal is to achieve a significant decrease in the number of seizures after DBS surgery, that is, a decrease that is statistically different from zero. For such an effect (Cohen’s *d* approximately 1) and an alpha risk of 5%, at least 10 subjects are needed for a power of 95% in a matched series comparison test. This means that, depending on the frequency and variability of our sample, the result will be positive if the seizure frequency decreases to 4 or 3 seizures per month.

Patients were screened for eligibility, followed by a 3-month baseline period during which the frequency and characteristics of their seizures were closely monitored. All patients signed an informed consent statement for the study at inclusion. After the PuM electrodes were implanted, patients were monitored regularly over the following 24 months (every 3 months). These visits include neurological/neurosurgical assessments, neuropsychological testing, and maintenance of a seizure diary (Supplementary Figure S1).

A neuropsychologist has been performing an extensive cognitive evaluation before inclusion and after 12 and 24 months after the start of PuM stimulation. The neuropsychological assessment includes the exploration of intellectual efficiency, long-term memory, short-term and working memory, executive functions, processing speed, motor skills, language, visual processing, and praxis. The patient has regularly filled out a seizure diary that has been checked at each visit. A standard EEG has been performed before inclusion and at 12- and 24-month follow-up.

A general assessment of neuropsychiatric comorbidities—Depression (NDDI-E scale) and anxiety (GAD 7 scale) (24)—the quality of life (QOLIE 31 scale) (25), seizure severity (NHS3 scale) (26), adverse event effect of AEDs (LAEP scale—Liverpool Adverse Events Profile) (27) have been performed at each visit (Supplementary Figure S1).

### Patients’ selection

Patients aged between 18 and 60 years suffering from focal or multifocal drug-resistant epilepsy not suitable for or after the failure of surgical intervention and deemed suitable for PuM stimulation after a multidisciplinary team meeting have been selected for the study. The target population included patients who failed previous neurostimulation techniques such as VNS (after at least 1 year of treatment or stopped earlier for seizure worsening) or anterior thalamic nucleus DBS (after 2 years of treatment or stopped earlier for seizure worsening). The PuM DBS may be indicated after failed cortical resection. To properly assess the primary outcome measure, it was considered that a minimal number of 4 seizures per month (for the last 3 months preceding inclusion) was necessary. To properly assess the secondary outcome (evaluation of neuropsychiatric and cognitive comorbidities), a QI >55 was considered mandatory. Patients presenting generalized epilepsy, contraindication to MRI, pregnancy, or presenting a history of attempted suicide in the 6 months before inclusion or a score ≥ 2 in item 10 of the Montgomery and Asberg Depression Scale (MADRS) were excluded. If the patient was treated with valproic acid for epilepsy, this drug would have been systematically stopped before surgery (28). This last criterion was added following the hemorrhagic event in P6.

### Primary and secondary endpoints

The hypothesis of the research was the following: chronic medial pulvinar (PuM) stimulation in patients with drug-resistant epilepsy will be associated with a significant decrease in seizure frequency as compared with seizure frequency previously experimented by the patient.

The study’s main objective was to obtain a significant seizure frequency reduction after stimulation of PuM compared to the seizure frequency calculated in the pre-implantation period of reference (9 to 12 months after implantation vs. 3 months before implantation). The objective was to show that PuM stimulation effectively reduces seizure frequency in the targeted population.

Some other relevant benefits observed under PuM DBS therapy have been analyzed as secondary endpoints: (1) comparing the quality of life at 1 and 2 years in relationship to the pre-stimulated period. (2) Assessing the psychiatric impact (depression and anxiety): evaluation of the psychiatric comorbidities is fundamental in drug-resistant epilepsy due to the high prevalence of these disorders (especially anxiety and depression) (8). Neuromodulation therapies have also been used in some psychiatric disorders with encouraging results (29). Monitoring with adapted scales (GAD 7 and NDDI) the symptoms related to anxiety and depression clarifies the global effects of PuM DBS, other than on seizure frequency. (3) Assessing the cognitive impact (neuropsychological examination). In the trial on anterior thalamic nucleus stimulation, the stimulated group was more likely to report memory problems as an adverse event (6). (4) Evaluation of the number of responders (identified as patients with >50% of seizure reduction). (5) Evaluating the number of seizure-free patients. (6) Safety assessment and possible side effects. (7) Evaluation of the change in seizure severity (NHS3 scale). Even if the seizure frequency has not been significantly modified, clinical experience suggests that patients could experience a better quality of life. A modification in seizure semiology, especially of disabling seizures (i.e., with loss of consciousness, fall, or long post-ictal phase), has potentially been observed.

### Surgical procedure

The surgical procedure was performed, as any DBS procedure for movement disorders or epilepsy in our institution, under general anesthesia with ROSA robotic assistance (Medtech, Zimmer Biomet) and intraoperative CT control (Airo, Moebus, and Strycker). The targeting of the Pulvinar medialis nucleus was based on direct visualization of 3D-T1- MP-RAGE sequences without any microelectrode recordings. The target was chosen to coincide with the location of the most distal contact of the SEEG electrode recording the medial pulvinar to be consistent with our study (21). The choice of the entry point and trajectory was determined by the individual anatomy of the patient and was in the vast majority of cases planned in double obliquity with a frontal precoronal lateral entry point. The degree of ventricular enlargement and the existence of other DBS hardware (ANT nucleus DBS when still in place) were crucial to determining the entry point and trajectory.

Robot registration was based on bony fiducials and Leksell G Frame (The mean rms value of registration should be below 0.75 mm). Intraoperative CT acquisition was performed after each lead implantation to check for proper positioning of the distal contact’s position.

The implantable pulse generator (IPG) implantation was performed on the same day, usually in the right subclavicular area [depending on the location of other IPG (ANT-DBS or VNS generator when still in place)].

The DBS hardware was as follows: four-contact chronic stimulation leads (3389) or more recently SenSight directional leads B33005 (Medtronic, Minneapolis, United States, CE marked), its extension kit DBS 37086 or more recently B34000 (60).

A postoperative CT scan was performed within 2–3 days after the procedure to double-check for the location of the lead and rule out any hemorrhagic complications. A comprehensive neurological examination was carried out immediately after surgery. Stimulation was switched on before the patient’s hospital discharge: 1/by choosing the first plot ideally located at the level of the PuM of the thalamus, based on the anatomical registration performed by the neurosurgeon. 2/systematically using the following stimulation paradigm: The parameters of stimulation are based on the SANTE trial parameters (30). The stimulation has been set at 145 Hz, 90 μs (pulse width), in bipolar mode (between the two best-located contacts), and in cycle mode with 5 min ON and 30 s OFF to start with. Delivered current was set according to the patient’s tolerance, generally starting at 1 V.

At follow-up visits, the investigator has been offered the possibility of augmenting PuM stimulation frequency and/or current intensity in case of clinical unresponsiveness. The stimulation has been left unchanged (cycle mode, bipolar) or switched to a monopolar (contact negative, case positive) to increase the volume of activated tissue around the active contact if necessary. Switching to continuous stimulation was considered if required and if the tolerance of cycle mode was good. The medications have been left unchanged as much as possible during the study, at least for the first 6 months, to rule out any potential confounding factors. However, some changes in anti-seizure drugs have been made and reported in Supplementary Table S1.

### Statistical analysis

All safety and efficacy variables have been summarized descriptively. Continuous variables have been described as means (standard deviation) or medians (quartiles) depending on the Gaussian distribution. Categorical variables have been described as frequency distributions (percentages). Comparisons used parametric or non-parametric tests according to the variables’ nature and distributions. Unless otherwise specified, statistical significance was defined as a *p*-value of <0.05.

Statistical analyses have been performed using R software. Demographic and baseline characteristics data have been summarized by the study group for all analyzed populations. Medical history findings, previous and concomitant medications, and other pertinent information have also been summarized.

Comparability with variables at baseline was verified using statistical methods for the comparison of paired samples according to the nature and distribution of the variables.

As reported in the CRF, the number of patients who prematurely dropped out has been summarized by reason. The change (expressed in %, relative change) of the number of seizures at 12-month follow-up (mean number per month during the previous 3 months) has been compared to the 3-month baseline period using the Wilcoxon signed-rank test (paired). Categorical variables (severity of epilepsy, quality of life, scores of depression, and anxiety) as estimated at V2 to V6 have been compared to those estimated at V0 using the Friedman test and/or Wilcoxon signed-rank test at each time point (V2, V3, V4, V5, and V6). The number of responders and seizure-free patients (> 50% of seizure reduction) has been calculated.

All adverse events (AEs) have been displayed on individual data listings with descriptions of full adverse events. The characteristics of adverse events were summarized by the study group.

The study group summarized the incidence of related adverse events (including possibly and probably related adverse events) with moderate or severe intensity.

When half of the expected number of patients reached 1-year follow-up, a preliminary analysis of the collected data according to the protocol was performed.

We reported here the results of DBS efficacy and safety in patients reaching 6 and 12 months of DBS. For neuropsychological examination, we analyzed scores on each subtest and test: (1) Scores on subtests of the Wechsler Intelligence Scale and the Wechsler Memory Scale are age-corrected data (In the normal population, these standard scores have a mean of 10 and an SD of 3) (31).

To exclude the test–retest practical effect reported in technical reference manuals of intellectual or memory scales, we defined a relevant change between the two evaluations as an increase or decrease of at least 3 points. Scores on additional tests were said to be normal or pathological, depending on the age group standard. We considered them changed if they moved from one category to another between two evaluations. Finally, we established that for each patient, the global percentage of subtests and tests (*n* = 29) improved, became stable, and decreased. We considered the NPS testing modified if 15% (>5/29) of modifications were noticed.

## Results

### Patients’ characteristics and pulvinar implantation

**Figure 1:**
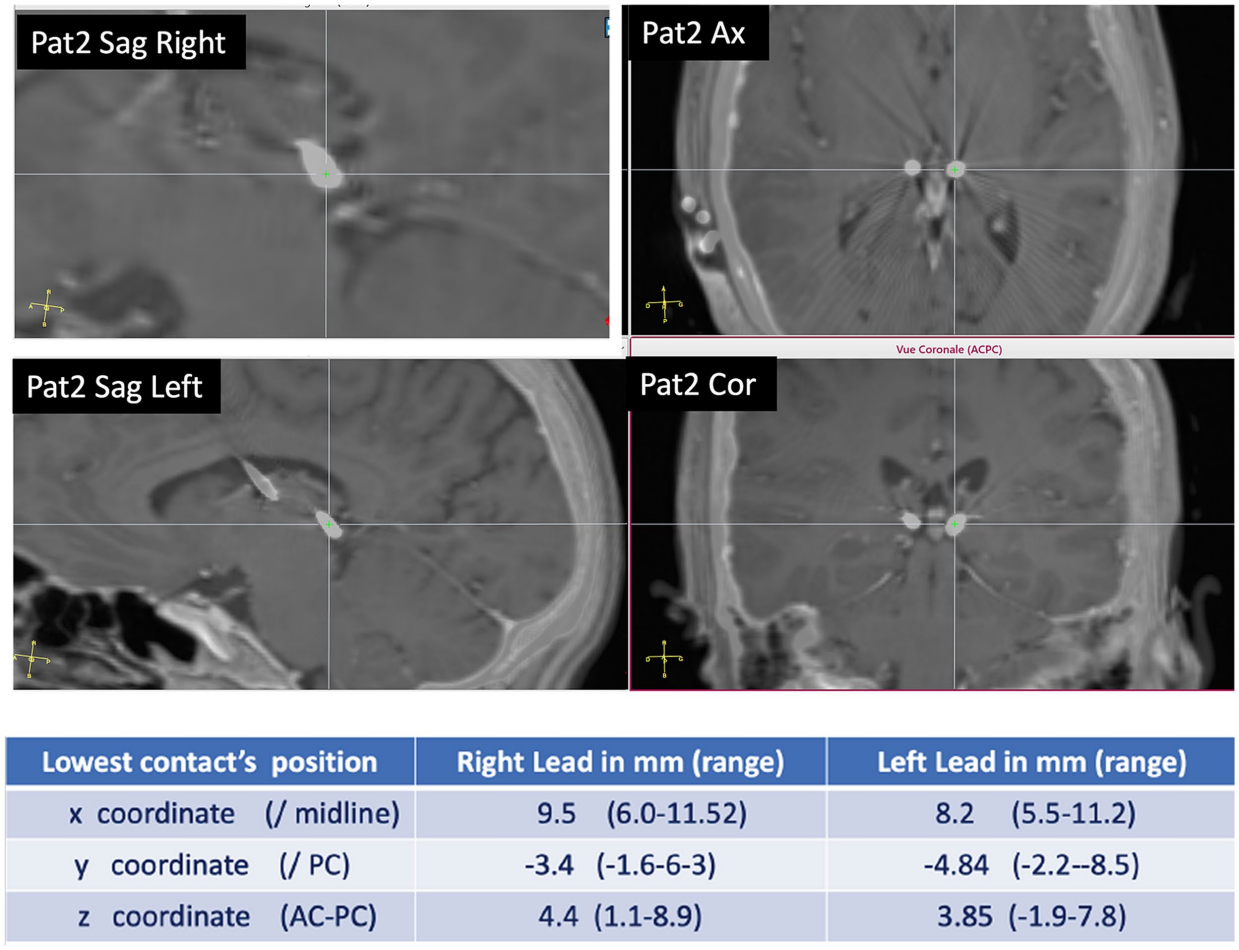
Pulvinar implantation: example of DBS electrode position in PuM (patient 2). In the table, the coordinates of the site of the more mesial contact in the DBS electrode are according to x, y, and z coordinates.

Five patients with available 6-month and 1-year follow-up have been analyzed. One patient (P3) has been analyzed only at a 6-month follow-up. All patients were implanted in the PuM (see Figure 1 for the electrodes’ visualization and electrode coordinates).

The patient’s mean age was 41.5 years (range 34–55 years). The main clinical characteristics are reported in Table 1. None of the patients had a previous epilepsy surgery, and all were contraindicated to surgery after video-EEG. Three patients (PT 1, PT3, and PT6) benefited from SEEG prior to DBS. In two cases (PT 1 and PT6), a pulvinar electrode was present during SEEG and was involved early in the recorded seizures. All patients had previously undergone VNS implantation, but there was no significant improvement, and three patients had the VNS implantation in ON mode before/during PuM implantation (Table 1). ANT-DBS was also in ON mode in three patients who were considered non-responders (Table 1).

**Table 1:** Patient characteristics, seizure frequency, and testing.

PT	Sex, age	Side	Seizure location	Etiology	Fam	VNS	ANT DBS	Resp 6M	Resp 12M	Seiz/M V1	Seiz/M V4	% Seiz change 6M	Seiz/M V6	% Seiz change 12M	Severity 12M	NPE 12M	QOLIE 12M	SAE
P1	F, 55	L	Post ins-op par	Crypto	N	Y	N	Y	Y	28.3	8.2	−71%	5.4	−81%	Impr	Impr	Stable	N
P2	F, 34	L	Temp+ (perisylv)	Rasmussen	N	Y	Y	Y	Y	7.6	3.4	−55%	2.6	−66%	Impr	Impr	Impr	Infection
P3	M, 35	L	Par-occ	Crypto	Y	Y (p)	N	N	NA	11	10.7	0%	NA	NA	NA	NA	NA	Death
P4	F, 40	B	Op	Infect	N	Y	N	N	N	12	7.7	−36%	9.1	−24%	Impr	Wors	Impr	N
P5	M, 47	B	Temp + (perisylv)	Crypto	N	N	Y	N	N	10.3	10.5	0%	9.1	−12%	Impr	Stable	Impr	N
P6	M, 38	B	Temp + (ins)	Crypto	N	N	Y	N	N	14.4	53.2	269%	11.8	−18%	Stable	Impr	Stable	Hemorrhage

Legenda: PT: patients, M: month, Fam: familiarity, Resp: responder, seiz: seizures, NPE: neuropsychological evaluation, QOLIE: quality of life quastionnaire, SAE: serious adverse event, post: posterior, ins: insula, par: parietal, temp: temporal, perisylv: perisylvian, occ: occipital, Op: opercular, crypto: cryptogenic, infect: infectious disease, N: non, Y: yes, p: paused, NA: not applicable, impr: improved, wors: worsened.

### Effect on seizure frequency and severity

**Figure 2:**
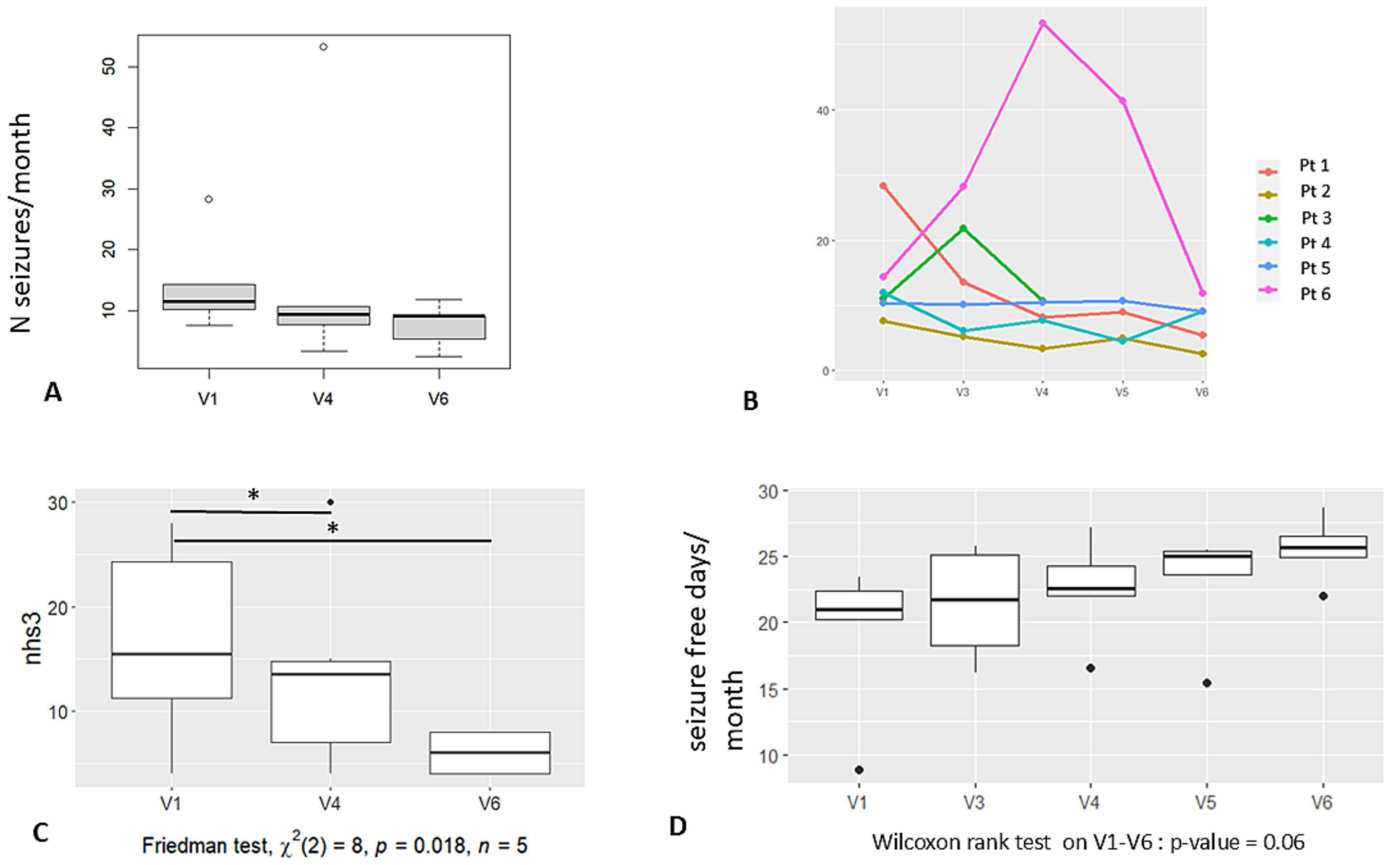
Graphic results—seizures. **(A)** Number of seizures per month at baseline (V1), at 6 months FU (V4), and at 12 months FU (V6). **(B)** Number of seizures per month according to each of the 6 patients and at each protocol visit. **(C)** Seizure severity (NHS3 score) calculated at V1, V4 and V6. Significant difference is noticed in scoring between V4, V6, and baseline. **(D)** Number of seizure-free days per month at each protocol visit. Legend: V1: baseline, V4: 6 months FU; V5: 9 months FU; V6: 12 months FU.

A trend toward seizure reduction was observed at 6 months, which became more evident at 1-year follow-up (Figure 2A) (primary outcome). However, this result did not reach significance in this small cohort of patients. In Table 1, the percentage of seizure reduction at 6 and 12 months per patient is reported, while Figure 2B shows graphically the number of seizures/month according to each patient.

The mean percentage of seizure frequency reduction for all patients at 1 year was −45%, while for the responder patients (P1 and P2), it was −78%.

Regarding seizure severity, we observed (5 out of 6 patients) a lowering of the global score of the NHS3 according to a shift toward less severe seizure semiology (in terms of LOA, time to recovery after the seizure, ground fall, etc.). The difference in seizure severity at V4 and V6 vs. V1 reached significance (Friedman test, x^2^, *p* < 0.02) (Figure 2C). The NHS3 scale was always performed by the same neurologist (FP) who interviewed the patient. Individual severity score modification at V6 is reported in Table 1.

We also observed a trend toward the augmentation of the number of seizure-free days/month (Figure 2D) without reaching significance (*p* = 0.06).

We further evaluated the number of responders (identified as patients with >50% of seizure reduction): two patients were responders at 6 months (2 out of 6) and at 1-year follow-up (2 out of 5) (Table 1). No seizure-free patients were observed.

### Impact on the quality of life and related measures

**Figure 3:**
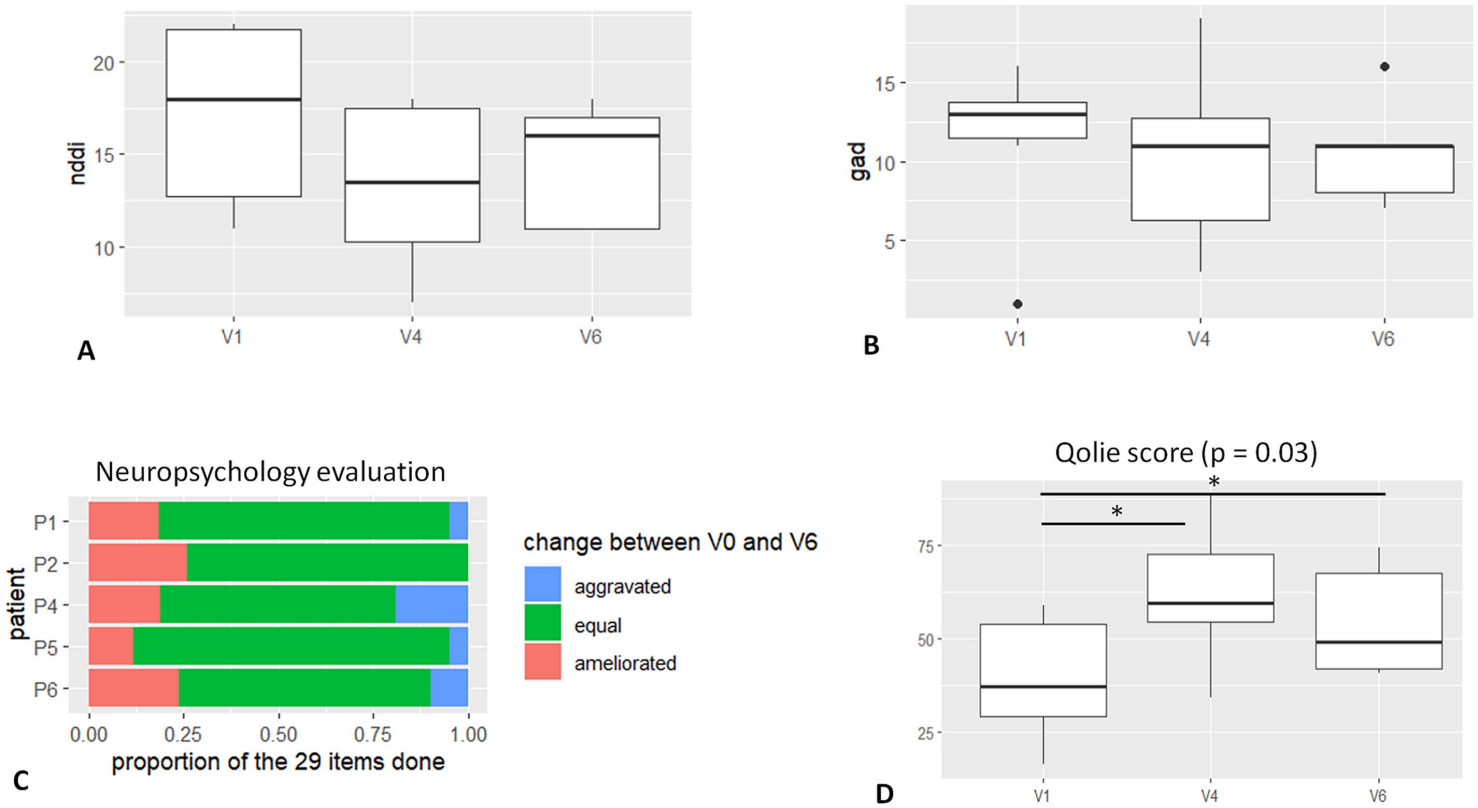
Graphic results—neuropsychology and quality of life. **(A)**. NDDI-E score at baseline (V1), 6 months FU (V4), and 12 months FU (V6). **(B)** GAD-7 score at baseline (V1), 6 months FU (V4), and 12 months FU (V6). **(C)** Table shows the percentage of ameliorated and worsted performance at neuropsychological evaluation. **(D)** QOLIE score at baseline (V1), 6 months FU (V4), and 12 months FU (V6). Significant difference is noticed in scoring between V4, V6, and baseline.

The quality of life at 6 months and 1-year follow-up resulted in significant improvement (*p* = 0.03) compared to the pre-stimulated period (Figure 3D). Regarding the different items of the QOLIE 31 testing, significance was found regarding “seizure apprehension,” “quality of life,” “emotional health,” and “energy and fatigue,” but not for “cognition,” “social aspects,” or “antiseizure medication.” Individual QOLIE improvement or stability at V6 is reported in Table 1.

One common observation of the patients after PuM stimulation was a state of “augmented reactivity” and/or more rapid responses to questions/actions. This has been reported but a clear measure of this effect is difficult to obtain.

Regarding the psychiatric impact (depression and anxiety), we did not find significant improvement in the related scales (Figures 3A,B). However, at 1-year FU, in four of five patients, the NDDI-E score was improved (mean 26%, range 8–50%), and GAD-7 was stable or improved (mean 20%, range 0–50%). One patient (P6 – DBS complicated with cerebral symptomatic hemorrhage) disclosed aggravation in NDDI-E and GAD-7 scores.

Regarding the stimulation’s cognitive impact (Figure 3C), the 29 scores obtained in tests and subtests remain largely stable (from 69 to 90%) for all patients. Three of five patients experience improvement in a number of scores. Improvement can represent up to 24% of the scores (P2). Cognitive domains concerned with improvement vary greatly from patient to patient. Improvement could occur regardless of baseline intellectual abilities, and improved scores do not necessarily correspond to strengths or weaknesses of baseline cognitive profile. A decrease of more than 15% in all scores is observed in one of the five patients. For this patient with higher normal intellectual efficiency, this decrease concerns speed processing and working memory scores, which moved from a high to a normal level. It occurred concomitantly with improved scores in verbal and visual reasoning tasks and does not affect a global intellectual level.

### Safety assessment and side effects

One patient (P3) died prematurely, possibly from a SUDEP 6 months after the beginning of the stimulation. One patient (P6) had a cerebral hemorrhage after DBS implantation with transitory left hemiparesis and dysphasia that almost completely recovered in the following months. One patient (P2) experienced a device infection that finally led to device removal (after 15 months) (Table 1).

Regarding side effects, paraesthesia has been reported as mild cephalalgia.

## Discussion

We reported preliminary results on six patients in an open clinical trial testing median pulvinar DBS in focal epilepsy. The final study includes 12 patients with a 2-year FU. Patients selected for the procedure are very complex cases of non-surgical drug-resistant epilepsy that failed to respond to VNS, and half of them also failed ANT-DBS. Regarding patient selection, based on the large cortical connections reported for pulvinar medialis [i.e., (15)] with temporal, frontal, parietal, and insulo-opercular regions and given its demonstrated role during focal seizures (16), we included patients with different and heterogeneous seizure locations. A common feature for inclusion was severe epilepsy, resistant to medications and VNS, with the majority of seizures involving loss of awareness.

The responder rate at 1-year follow-up is approximately 40% (two out of five patients), somewhat lower than the 54% reported for ANT-DBS (6). However, we noted a significant reduction in seizure severity as indicated by NHS3 scores and a trend toward increased seizure-free days per month. In fact, in both responder patients and in two out of three non-responder patients, the severity of seizures decreased, even though the seizure frequency did not significantly change.

In two patients (P3 and P6), we observed an initial increase in seizure frequency. Regarding P6, it could be related to the effects of cerebral hemorrhage following DBS implantation, with normalization occurring after recovery. In P3, aggravation in seizure frequency could be linked to the initial anti-seizure medication modification, which was necessary due to poor compliance (cfr suppTab), and later resolved. Moreover, subsequent amelioration in seizure frequency could be linked to the effect on neuromodulation through time.

Similar to other neuromodulation techniques, such as the VNS (9), we observed a significant improvement in the quality of life in the majority of patients (Figure 3D; Table 1). The first data on this small cohort of patients did not show aggravation in the scores of anxiety and depression but mostly a trend to amelioration (Figures 3A,B). A very promising result is the amelioration in neuropsychological testing in 3 out of 5 patients with 1-year follow-up, which is rarely seen in the adult population with drug-resistant epilepsy (Figure 3C). Pulvinar medialis is not involved in the Papez circuit as the ANT, so its stimulation might have less implication in memory/cognition alteration, as was reported in the first studies on ANT-DBS (6). Nonetheless, pulvinar medialis is a high-order nucleus (32), connected with multiple associative areas in the brain, and plays an essential role in attention (33, 34) and goal-oriented behavior (35). Its connectivity might improve cognitive skills and may be linked to the reported “augmented reactivity” in some patients. However, an attentive neuropsychological examination is deemed necessary.

Pulvinar stimulation, with DBS or RNS, has been reported in a few studies. Burdette et al. (22) reported very promising results in three patients, all responders, with both pulvinar and cortical RNS, for posterior quadrant epilepsy. Interestingly, they also noted mild cognitive improvement. Additionally, one case of pulvinar stimulation in Ohtahara syndrome has been described (36), as well as one case in occipital epilepsy with good results (37), and another in a patient with temporal plus epilepsy (38).

In the literature, generally, pulvinar stimulation seems to be reserved for “posterior” epilepsy. In fact, the lateral pulvinar (PuL) is crucial for visual attention and spatial processing, selectively transmitting visual information between the primary visual cortex (V1) and higher-order areas such as V4. Its extensive connectivity supports top-down attentional control and synchronization of activity across the visual cortex (39, 40). However, the pulvinar is the largest nucleus in the thalamus, and important differences exist between its lateral and medial parts (41, 42). According to functional anatomy, the PuM plays an important role in emotional processing and attention modulation (33), integrating sensory and emotional information through connections with the limbic system (43, 44), such as the amygdala and hippocampus, as well as the prefrontal cortex (43). The medial pulvinar also connects with the posterior parietal cortex, which is involved in spatial attention, and the insula, facilitating the integration of emotional states and interoceptive awareness (43).

To summarize, it should be noted that the stimulation of PuM, reported in our study, was proposed according to the known largest PuM connections (45) and not specifically to connections to “visual” areas as is the case of PuL.

In patients already receiving VNS and/or ANT-DBS, it is challenging to determine whether the reported clinical improvement is solely due to PuM DBS or a synergistic/delayed effect from the other neuromodulation techniques. In our cohort, the two responder patients experienced a marked improvement immediately following the addition of PuM DBS, which dramatically altered their clinical condition. This significant change is unlikely to be attributed solely to a delayed effect of previous stimulations, although a synergistic effect cannot be ruled out.

From a surgical perspective, targeting the PuM is challenging in several respects. First, half of the patient cohort had another DBS hardware in place, which allows little flexibility as to the choice of the entry point, given the presence of pre-existing ANT-DBS lead wires subcutaneously that should not be damaged. In addition, the double obliquity trajectory does not offer much latitude as to the entry point because of a certain degree of ventricular enlargement that makes it compulsory to place the entry point quite lateral to reach the very medial part of the pulvinar. A parietal approach or purely orthogonal approach could be resorted to but may not be advisable on several grounds because it also comes with technical constraints and risks. Finally, in some patients, a tradeoff must be found between implanting the implantable pulse generator (IPG) on the contralateral subclavicular, thus making a new scar potentially untoward for cosmetic reasons but in a healthy anatomical area, and using a previously operated-on area with a pre-existing scar with some increased risk of healing problems or infection.

We faced three serious adverse events (infection, bleeding, and SUDEP). All these complications have been reported in previous DBS studies (30) and do not appear specific to this target.

A more detailed analysis according to the etiology or epilepsy location is not possible at this stage.

## Conclusion

In conclusion, we felt it was essential to publish the preliminary results of this study, given the current enthusiasm for PuM and its stimulation in epilepsy (22, 36, 38, 46). We observed a trend toward a positive effect on seizure severity, quality of life, and cognition. While the impact on seizures is more variable, two patients responded well. The full report on the 12-patient study after a 2-year follow-up will allow us to confirm these trends in the future.

## Data Availability

The datasets presented in this article are not readily available because ongoing clinical trial. Data are confidential until the end of the study. Requests to access the datasets should be directed to francesca.pizzo@ap-hm.fr.
