# Supplementary material for: Medial pulvinar stimulation for focal drug-resistant epilepsy: interim 12-month results of the PULSE study
Source: Front Neurol. 2024 Dec 10;15:1480819. doi: 10.3389/fneur.2024.1480819 (PMC11667892; doi:10.3389/fneur.2024.1480819)
Supplement: Supplementary file 1 [file Data_Sheet_1.pdf]

Patient	between V0-V1	V1	between V1-V2	V2	V3	between V3-V4	V4	V5
1	no change	no change	no change	no change	no change	stop and restart clobazam	no change	no change
2	no change	no change	no change	no change	no change	no change	no change	BRV from 100 to 125 mg/die
3	no change	Clobazam from 10 to 5 mg/die; LEV from 3000 to 2750 mg/die	VPA from 750 to 1000 mg/die, stop FLB 1200 mg/die	no change	CBZ from 600 to 800 mg/die; CLB from 30 to 25 mg/die	Clobazam from 25 o 20 mg/die	no change	no change
4	Start CNB up to 150 mg/die	no change	no change	no change	no change	no change	no change	Stop CLB 5 mg/die
5	no change	no change	no change	no change	no change	no change	no change	no change
6	no change	no change	VPA from 500 to 750 mg/die ; STOP CLB 25 mg/die; CBZ from 1600 to 1200 mg/die; VGB from 1000 to 750 mg/die	no change	no change	no change	no change	no change
