# Supplementary figures and images for: Medial pulvinar stimulation for focal drug-resistant epilepsy: interim 12-month results of the PULSE study
Source: Front Neurol. 2024 Dec 10;15:1480819. doi: 10.3389/fneur.2024.1480819 (PMC11667892; doi:10.3389/fneur.2024.1480819)

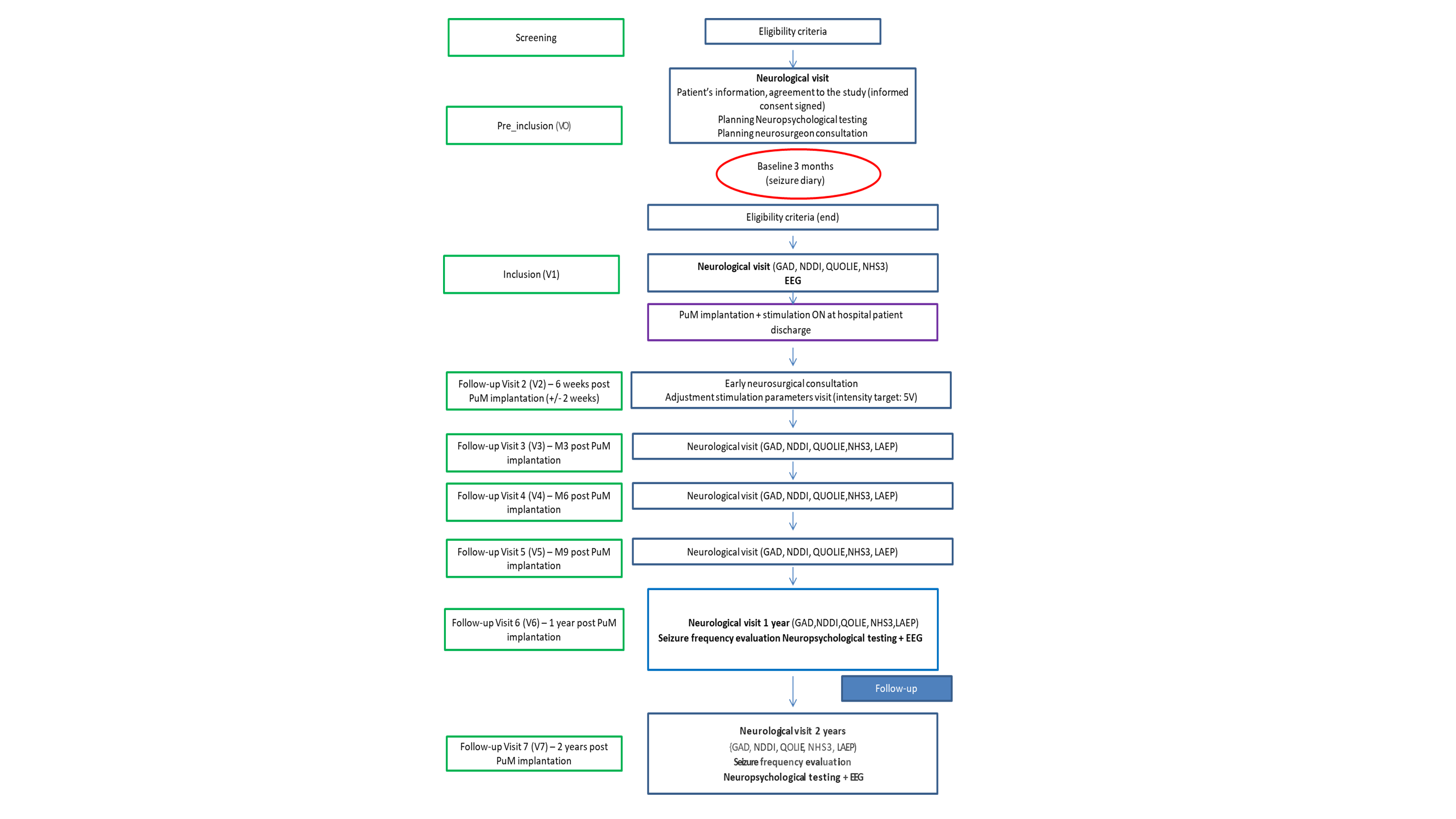

Supplement: Supplementary file 2 [file Image_1.tif]
